# Measurable residual disease of canonical versus non-canonical *DNMT3A*, *TET2*, or *ASXL1* mutations in AML at stem cell transplantation

**DOI:** 10.1038/s41409-021-01407-6

**Published:** 2021-07-15

**Authors:** Madlen Jentzsch, Juliane Grimm, Marius Bill, Johannes Küpper, Donata Backhaus, Dominic Brauer, Julia Schulz, Georg-Nikolaus Franke, Vladan Vucinic, Dietger Niederwieser, Uwe Platzbecker, Sebastian Schwind

**Affiliations:** Medical Clinic and Policlinic 1, Hematology, Cellular Therapy and Hemostaseology, University of Leipzig Medical Center, Leipzig, Germany

**Keywords:** Translational research, Acute myeloid leukaemia

**To the Editor**,

Mutations affecting the genes *DNMT3A*, *TET2*, and *ASXL1*—commonly referred to as DTA mutations—belong to the founding mutations in acute myeloid leukemia (AML) but usually are not capable to initiate the disease by themselves [1]. While *TET2* mutations do not have a common hotspot, mutations in *DNMT3A* and *ASXL1* frequently occur at specific positions (*DNMT3A* in R882 and *ASXL1* as G646fs*12, henceforward referred to as “canonical”). DTA mutations seem to grant a proliferation advantage to hematopoietic progenitor cells, leading to their expansion in otherwise healthy individuals over time and also to a rapid bone marrow repopulation after chemotherapy in AML patients [2]. Thus, their detection in complete remission can be attributed to either measurable residual disease (MRD) with associated higher relapse risk or a pre-leukemic clone with a much lower risk of AML reoccurrence. Analyses that studied the dynamic mutation burden during AML disease course described a lower probability of DTA mutations clearance after chemotherapy compared to mutations in genes reflecting secondary genetic events, such as *FLT3* or *NRAS* [3, 4]. Furthermore, while DTA mutations often persisted at high variant allele frequencies (VAFs) in AML remission, these aberrations mostly do not lead to increased relapse rates within the follow-up time of the respective studies [4, 5]. On the other hand, when mutation burden and clinical course were correlated in single individuals, at least in some patients, *DNMT3A* mutations paralleled the dynamics of the *NPM1* mutation and clinical disease burden [6]. However, in studies not considering the specific mutation type of DTA mutations, a clinical utility for MRD detection could not be shown after induction therapy [4, 7] or at the end of treatment [4]. Subsequently, a limited relevance as MRD markers in AML patients was suggested which led to the exclusion of DTA mutations from most MRD studies [7, 8].

Although randomized trials are lacking, there is evidence that allogeneic hematopoietic stem cell transplantation (HSCT) may improve outcomes in patients who remain MRD positive after intensive chemotherapies [3, 9], with the caveat of inferior outcomes for MRD- positive compared to MRD-negative patients after HSCT [8, 10, 11]. Two studies impressively pointed out that the detection of persisting gene mutations at HSCT associates with adverse clinical outcomes [8, 10]. However, *DNMT3A* mutations were excluded in one analysis [8] and both studies did not report on *ASXL1* mutations—most likely due to the difficulties to detect insertions at codon 646 by NGS technology. Due to restricted patient numbers, the remaining data on *TET2* and *DNMT3A* were not sufficient to draw clinical conclusions prior to HSCT [8, 10]. Thus, the possibilities to define risk stratification prior to allogeneic HSCT in patients harboring DTA mutations have not yet been evaluated. One study analyzed the impact of detectable DTA mutations after HSCT but could not draw explicit conclusions [12], and none of the aforementioned studies analyzed canonical and non-canonical DTA mutations separately.

We analyzed 68 AML patients who harbored at least one DTA mutation at diagnosis and received an allogeneic HSCT at a median age of 64.1 (range 34.7–75.3) years. Patients’ characteristics are given in Supplementary Table S1. Written informed consent was obtained from all patients in accordance with the Declaration of Helsinki. Sixty-three patients had one mutated DTA gene (37 affecting *DNMT3A*, 13 affecting *TET2,* and 13 affecting *ASXL1*), four patients had two mutated DTA genes (three with mutated *DNMT3A* and mutated *TET2*, and one with mutated *TET2* and *ASXL1*) and one patient harbored mutations in all three genes. Of the detected mutations in *ASXL1* and *DNMT3A*, 3/15 and 16/41 were non-canonical, not affecting the respective G646 or R882 hotspot regions. Frequent co-mutations and the distribution of DTA mutated patients within the ELN2017 risk groups are shown in Fig. 1A, B. There were no outcome differences between patients harboring a *DNMT3A*, *TET*2, or *ASXL1* mutation at diagnosis (Fig. S1).

**Fig. 1:**
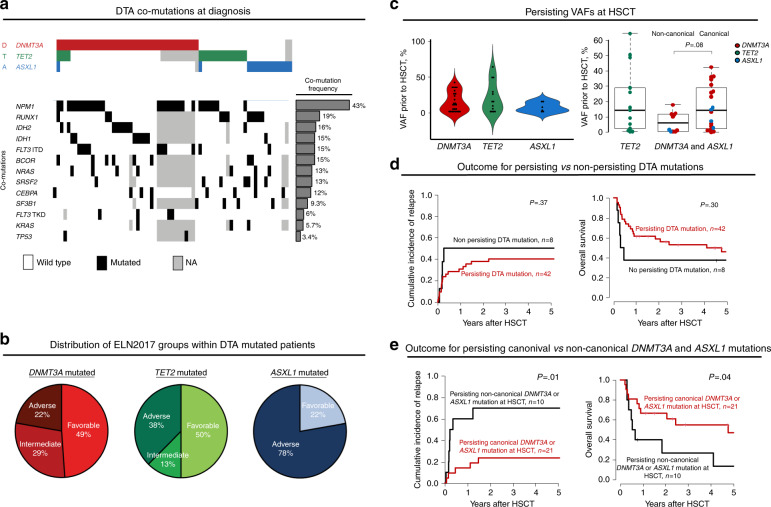
Analysis of DTA mutations at diagnosis and as MRD markers prior to allogeneic HSCT. **A** Co-mutational pattern and (**B**) Distribution of the three ELN2017 risk groups within *DNMT3A*, *TET2*, and *ASXL1* mutated AML patients at diagnosis. **C** Overview of VAF levels of persisting *DNMT3A*, *TET2*, and *ASXL1* mutations and comparison of persisting non-canonical vs canonical *DNMT3A* or *ASXL1* mutations at HSCT. **D** Cumulative Incidence of Relapse and Overall Survival according to persisting vs non-persisting DTA mutations at HSCT (*n* = 50). **E** Cumulative Incidence of Relapse and Overall Survival according to the presence of a non-canonical vs a canonical mutation in patients with persisting *DNMT3* or *ASXL1* mutations.

Analyzing patients with available paired samples at diagnosis and HSCT (for applied assays and assay sensitivity see Table S2 and Figs. S2, S3), the majority of patients (84%) had persisting DTA mutations. *DNMT3A* mutations persisted in 25/29, *TET2* in 13/14, and *ASXL1* in 6/10 patients in remission at HSCT. Of the patients with multiple DTA mutations, mutations at HSCT concordantly persisted in 2 of 3 patients. In one patient an *ASXL1* mutation was not detectable while a *TET2* mutation persisted at a VAF of 1.1%. In *DNMT3A* and *ASXL1* mutated patients, there was no difference regarding the frequency of persisting canonical *vs* non-canonical mutations (17/19 vs 8/10, *P* = 1 and 4/7 *vs* 2/3, *P* = 1, respectively). Of the analyzed non-canonical mutations in *DNMT3A* 10% were frameshift, 60% were missense, and 30% were nonsense, while in *ASXL1* all non-canonical mutations were nonsense (Table S3). Overall, DTA mutations persisted at HSCT at highly variable VAFs with a median of 11.7% (range 0.4–42.5) for *DNMT3A*, 15.2% (range 0.3–64.5) for *TET2* and 5.1% (range 0.4–16.1) for *ASXL1* mutations. Non-canonical *DNMT3A* and *ASXL1* mutations tended to persist at lower VAFs compared to canonical mutations (*P* = .08, Fig. 1C). Characteristics of canonical compared to non-canonical *DNMT3A* and *ASXL1* mutations at HSCT are given in Table S4.

In line with the general opinion in the field, we did not observe an association of persisting DTA mutations at HSCT with a distinct cumulative incidence of relapse (CIR, *P* = .37) or overall survival (OS, *P* = 0.30, Fig. 1D). However, in *DNMT3A* or *ASXL1* mutated patients, the persistence of a non-canonical mutation associated with significantly higher CIR (*P* = 0.01) and shorter OS (*P* = 0.04) compared to the persistence of canonical mutations (Fig. 1E) suggesting that they—in contrast to canonical mutations—might have a different clinical value as they seem to be able to detect AML MRD.

As studies regarding the utility of MRD markers at HSCT for DTA mutated patients have not been published, we correlated available MRD data at HSCT for our patient cohort with outcome adapting established MRD assays in our institution (Supplementary Material) [11]. Including non-canonical *DNMT3A* and *ASXL1* mutations MRD to the MRD information derived from *NPM1* mutation and *BAALC*/*ABL1* and *MN1*/*ABL1* copy numbers MRD led to improved Bayesian Information Criterion (BIC) models for CIR and OS prediction (Table S5). Combining all five MRD markers, DTA mutated patients with at least one positive MRD marker at HSCT had a higher CIR (*P* = 0.002) and shorter OS (*P* = 0.001, Fig. S4). MRD positivity retained its prognostic power in multivariate analyses for CIR and OS, (Table S6) while also the number of positive MRD markers (no vs one vs ≥ two positive MRD markers) correlated with stepwise worse outcomes after HSCT (Fig. S4).

With a median age at HSCT of 64.7 years our study reflects a common AML patient population. Median follow up was 5.0 years from HSCT, a time interval in which the majority of AML patients suffer their relapse. However, the study’s limitations are its’ restricted patient numbers, as well as its retrospective nature with the need for confirmation in larger clinical trials.

In conclusion, our data demonstrate that overall, the detection of DTA mutations at diagnosis and prior to allogeneic HSCT does not associate with adverse outcomes, including late events after HSCT. However, non-canonical *DNMT3A* and *ASXL1* mutations seem to persist at lower VAF levels and associate with worse outcomes compared to the persistence of canonical mutations, challenging the current paradigm that all DTA mutations are unsuitable for MRD evaluation. Including non-canonical *DNMT3A* and *ASXL1* mutations in the armory of useful MRD markers may help to improve the risk stratification of DTA mutated patients.

## Supplementary information

Supplementary Material-clean
