# Supplementary material for: Measurable residual disease of canonical versus non-canonical DNMT3A, TET2, or ASXL1 mutations in AML at stem cell transplantation
Source: Bone Marrow Transplant. 2021 Jul 15;56(10):2610–2. doi: 10.1038/s41409-021-01407-6 (PMC8486652; doi:10.1038/s41409-021-01407-6)
Supplement: Supplementary file 1 — Supplementary Material-clean [file 41409_2021_1407_MOESM1_ESM.docx]

**SUPPLEMENTARY INFORMATION**

**Measurable Residual Disease of canonical versus non-canonical *DNMT3A*, *TET2*, or *ASXL1* mutations in AML at stem cell transplantation**

Jentzsch *et al*.

**Supplementary Methods**

**Induction therapy protocols of patients in the outcome set**

One patient was treated within the PKC wild type trial (ClinicalTrials.gov Identifier: NCT03512197) and with CPX-351, respectively. Four patients received standard 7+3 chemotherapy, and two patients were treated within the Quantum first trial (ClinicalTrials.gov Identifier: NCT02668653). All other patients were treated according to the acute myeloid leukemia (AML) 2002 study (OSHO #061, patients younger than 60 years at diagnosis,^1^ n=22) or according to the AML 2004 study (OSHO #069, patients older than 60 years at diagnosis,^2^ n=38).

**Prevention of graft-versus-host disease**

Prevention of graft-versus-host disease (GvHD) was different according to the conditioning regimes used. All patients receiving myeloablative (MAC) or reduced intensity (RIC) conditioning were treated with cyclosporine A (CyA), starting intravenously with 5 mg/kg body weight (BW) in two daily doses from day -1. Blood levels of CyA were measured from day 0 and doses were adjusted for target trough levels of 200 ng/ml. Patients also received methotrexate 15 mg intravenously on days +1, +3, +6, and +11 after hematopoietic stem cell transplantation (HSCT). Furthermore, patients with an unrelated donor additionally received *in vivo* T-cell depletion with thymoglobulin 2 mg/kg BW per day on days -3 to -1.

All patients with non-myeloablative (NMA)-HSCT received a starting dose of 5 mg/kg BW CyA in two daily doses from day -1. Blood levels of CyA were measured from day 0 and doses were adjusted for target trough levels of 200 ng/ml. Additionally, patients with NMA conditioning received mycophenolate mofetil (MMF) 3 g per day in three daily doses if receiving unrelated HSCT or 2 g per day in two daily doses if receiving related HSCT. CyA was reduced starting on day +84 or day +180 following related or unrelated HSCT, respectively, and MMF was stopped at day +28 following related HSCT and tapered from days +40 to +96 following unrelated HSCT.^8^ For all patients after MAC-, RIC-, or NMA-HSCT, immunosuppression was prolonged or extended with systemic steroids in cases of GvHD (grade > 2 according to Glucksberg grading system^3^) or rapidly reduced in patients who relapsed (≥ 5% blasts in bone marrow). Patients were evaluated for incidence of acute GvHD (GvHD) and chronic GvHD, using established criteria of the Glucksberg grading system.^3^ Requirement for acute GvHD was engraftment while requirement for chronic GvHD was engraftment and survival for at least 100 days after HSCT.

**Definition of complete remission**

Complete remission (CR) was defined as the presence of <5% of blasts in bone marrow (BM), neutrophils >1.0 x 10^9^/L, platelets >100 x 10^9^/L, absence of blasts with Auer rods, independence of blood transfusion and no extramedullary disease.^4^ CR with incomplete peripheral recovery (CRi) was defined as CR with platelets <100 x 10^9^/L or neutrophils <1.0 x 10^9^/L. In patients receiving allogeneic HSCT, the presence of CR or CRi was confirmed within 28 days prior to HSCT by bone marrow and peripheral blood analysis.

**Cytogenetics and molecular markers**

Diagnostic cytogenetic analyses were performed centrally using standard techniques of banding and *in situ* hybridization. The mutation status of the genes CCAAT/enhancer-binding protein alpha (*CEBPA*)*,* nucleophosmin 1 (*NPM1), DNMT3A* R882 hotspot, as well as the presence or absence of an internal tandem duplications in the *FLT3* gene (*FLT3*-ITD) were evaluated as previously described.^5,6^ For patients with material available (n=52), the mutation status of 54 genes included in the TruSight Myeloid Sequencing Panel (Illumina, San Diego, CA, USA) was evaluated using next generation sequencing (NGS) at diagnosis as previously described.^7,8^ *ASXL1* mutations at codon 646 were validated applying a proof-reading polymerase based Sanger sequencing approach.^9^ Previously described germline *DNMT3A, ASXL1*, and *TET2* variants were excluded from the analysis. Patients were grouped according to the 2017 European LeukemiaNet (ELN2017) recommendations.^10^

**Evaluation of *DNMT3A*, *TET2*, and *ASXL1* mutation status at HSCT**

In patients with material available up to 28 days prior to HSCT in CR/CRi, peripheral blood or bone marrow was collected for evaluation of the known DTA mutations adapting targeted amplicon sequencing on the MiSeq platform using a custom panel as previously described^8^ and/or mutation-specific primer/probes ddPCR assays. All patients with a negative test result in NGS (limit of detection 3%) were additionally analyzed using ddPCR to detect DTA mutations at MRD level. Supplementary Table S2 shows the evaluated canonical *DNMT3A* and *ASXL1* mutations with their respective primer/probe design, PCR conditions, and sensitivity. For patients with non-hotspot mutations, patient-specific primer/probe pairs were designed and pre-HSCT material measured with diagnostic material used as positive controls (see Supplementary Table S3 for analyzed mutations per patient). Supplementary Figure S2 shows dilution series for four exemplary mutations which demonstrate a linear dilution down to a mutant/wild type ratio of 0.0001.

**Additional MRD assessment in CR/CRi prior to allogeneic HSCT**

Additional MRD evaluation was performed using established ddPCR assays for the MRD targets mutated *NPM1*,^11^ *BAALC/ABL1* copy numbers,^12^ and *MN1/ABL1* copy numbers.^13^ Thresholds for MRD-positivity were adapted as previously published (*i.e.* > 0.01% or ≥ 3 positive droplets for *NPM1*, ≥ 0.14 *BAALC*/*ABL1* copies or ≥ 0.30 *MN1*/*ABL1* copies).^11–13^

Since *NPM1* mutations are the most frequent co-mutations (43% in this cohort; Figure 1A) in DTA mutated AML patients and represent an established MRD marker, they certainly stand out as the single most important MRD marker. The remaining *NPM1* wild-type patients may not be covered with techniques reaching MRD level detection as easily. However, *BAALC*/*ABL1* or *MN1*/*ABL1* expression based MRD analyses have been shown to be useful tools to detect early relapse after HSCT independent of the underlying mutation patterns.^11–14^

**Correlation of NGS and ddPCR results**

In a total of 46 patient samples (n=17 at diagnosis and n=29 prior to HSCT) the DTA mutations (*DNMT3A*, n=18; *TET2*, n=17; *ASXL1*, n=11) were assessed using both methods. For all genes, a good correlation between NGS and ddPCR results could be observed (Supplementary Figure S3).

**Statistical analyses**

All statistical analyses were performed using the R statistical software platform (version 4.0.2).^15^ OS was calculated from HSCT until death from any cause. The competing risks cumulative incidence of relapse (CIR) and NRM were calculated from HSCT to relapse or death, respectively, using the Fine and Gray method.^16^ Associations with baseline clinical, demographic, and molecular features were compared using the Kruskal-Wallis-Test and Fisher’s exact tests for continuous and categorical variables, respectively. Survival estimates were calculated using the Kaplan-Meier method and groups were compared using the log-rank test.

**Multivariate analyses**

We constructed multivariable proportional hazard model for cumulative incidence of relapse (CIR), and overall survival (OS) to evaluate the impact of MRD status prior to HSCT by backward adjusting for other variables. The following variables were considered for multivariable analyses: sex, disease origin (*de novo* vs secondary), European LeukemiaNet (ELN) risk, mutation status of the tyrosine kinase domain of the *FLT3* gene (*FLT3*-TKD), age at HSCT, disease status at HSCT (MRD^neg^ *vs* MRD^pos^), number of remission (first *vs* second), cytomegalovirus (CMV) status of recipient and donor (high-risk [+/-] *vs* all others), donor type (matched related *vs* matched unrelated *vs* mismatched unrelated), and sex of the donor (female into male *vs* all others). Of these, variables significant at α=.10 in univariable analyses were considered for multivariable analyses. For all endpoints, hazard/odds ratios with their corresponding 95% confidence intervals are indicated for every significant prognostic factor of the final model.

**Supplementary Results**

**Patients’ characteristics according to canonical or non-canonical *DNMT3A* and *ASXL1* mutations at HSCT**

Clinical, genetic, and molecular information at diagnosis according to the persistence of a canonical *vs* non-canonical *DNMT3A* or *ASXL1* mutations at HSCT are shown in Supplementary Table S4.

**Significance of MRD status at HSCT in DTA mutated patients**

When comparing the Bayesian Information Criterion (BIC) of MRD analysis at HSCT using a model with three MRD markers (*NPM1* mutation and *BAALC/ABL1*, and *MN1/ABL1* expression) to the model with five markers (additionally including non-canonical *DNMT3A* and *ASXL1* mutations), the 5-marker model resulted in lower BIC levels (Supplementary Table S5) and thus, represents the better statistical model. Subsequently, all MRD analyses were performed using the 5-marker model and patients with at least one positive MRD marker were regarded as MRD-positive. In this cohort of patients harboring DTA mutations at diagnosis, apart from the presence of at least one positive MRD marker at HSCT, also a stepwise higher CIR (*P*=.001, Supplementary Figure S4C) and shorter OS (*P*=.004, Supplementary Figure S4D) was observed according to the number of positive MRD markers at HSCT (no *vs* one *vs* ≥ two positive MRD markers).

**Supplementary References**

1 Büchner T, Schlenk RF, Schaich M, Doḧner K, Krahl R, Krauter J *et al.* Acute Myeloid Leukemia (AML): Different treatment strategies versus a common standard arm - Combined prospective analysis by the German AML Intergroup. *J Clin Oncol* 2012; **30**: 3604–3610.

2 Niederwieser D, Hoffmann VS, Pfirrmann M, Al-Ali HK, Schwind S, Vucinic V *et al.* Comparison of Treatment Strategies in Patients over 60 Years with AML: Final Analysis of a Prospective Randomized German AML Intergroup Study. [abstract]. In: *Blood*. 2016, p 1066.

3 Glucksberg H, Storb R, Fefer A, Buckner CD, Neiman PE, Clift RA *et al.* Clinical manifestations of graft-versus-host disease in human recipients of marrow from HL-A-matched sibling donors. 1974; : 295–304.

4 Döhner H, Estey EH, Amadori S, Appelbaum FR, Büchner T, Burnett AK *et al.* Diagnosis and management of acute myeloid leukemia in adults: Recommendations from an international expert panel, on behalf of the European LeukemiaNet. *Blood* 2010; **115**: 453–474.

5 Bill M, Jentzsch M, Grimm J, Schubert K, Lange T, Cross M *et al.* Prognostic impact of the European LeukemiaNet standardized reporting system in older AML patients receiving stem cell transplantation after non-myeloablative conditioning. *Bone Marrow Transplant* 2017; **52**: 932–935.

6 Schmalbrock LK, Bonifacio L, Bill M, Jentzsch M, Schubert K, Grimm J *et al.* Prognostic relevance of DNMT3A R882 mutations in AML patients undergoing non-myeloablative conditioning hematopoietic stem cell transplantation. *Bone Marrow Transplant* 2018; **53**: 640–643.

7 Jentzsch M, Bill M, Grimm J, Schulz J, Schuhmann L, Brauer D *et al.* High expression of the stem cell marker GPR56 at diagnosis identifies acute myeloid leukemia patients at higher relapse risk after allogeneic stem cell transplantation with the CD34+/CD38- population. *Haematologica* 2020; **105**: e507.

8 Grimm J, Bill M, Jentzsch M, Beinicke S, Häntschel J, Goldmann K *et al.* Clinical impact of clonal hematopoiesis in acute myeloid leukemia patients receiving allogeneic transplantation. *Bone Marrow Transplant* 2019; **54**. doi:10.1038/s41409-018-0413-0.

9 Metzeler KH, Becker H, Maharry K, Radmacher MD, Kohlschmidt J, Mrózek K *et al.* ASXL1 mutations identify a high-risk subgroup of older patients with primary cytogenetically normal AML within the ELN Favorable genetic category. *Blood* 2011; **118**: 6920–6929.

10 Döhner H, Estey E, Grimwade D, Amadori S, Appelbaum FR, Ebert BL *et al.* Diagnosis and management of AML in adults: 2017 ELN recommendations from an international expert panel. *Blood* 2017; **129**: 424–447.

11 Bill M, Grimm J, Jentzsch M, Kloss L, Goldmann K, Schulz J *et al.* Digital droplet PCR-based absolute quantification of pre-transplant NPM1 mutation burden predicts relapse in acute myeloid leukemia patients. *Ann Hematol* 2018; **97**: 1757–1765.

12 Jentzsch M, Bill M, Grimm J, Schulz J, Goldmann K, Beinicke S *et al.* High BAALC copy numbers in peripheral blood prior to allogeneic transplantation predict early relapse in acute myeloid leukemia patients. *Oncotarget* 2017; **8**: 87944–87954.

13 Jentzsch M, Bill M, Grimm J, Schulz J, Beinicke S, Häntschel J *et al.* Prognostic Impact of Blood MN1 Copy Numbers Before Allogeneic Stem Cell Transplantation in Patients With Acute Myeloid Leukemia. *HemaSphere* 2019; **3**: e167.

14 Jentzsch M, Grimm J, Bill M, Brauer D, Backhaus D, Schulz J *et al.* Prognostic relevance of remission and measurable residual disease status in AML patients prior to reduced intensity or non-myeloablative allogeneic stem cell transplantation. *Blood Cancer J* 2021; : in press.

15 R Development Core Team. R: A language and environment for statistical computing. Vienna, Austria. 2017. doi:R Foundation for Statistical Computing, Vienna, Austria. ISBN 3-900051-07-0, URL http://www.R-project.org.

16 Gray RJ. A Class of K-Sample Tests for Comparing the Cumulative Incidence of a Competing Risk. *Ann Stat* 1988; **16**: 1141–1154.

**Supplementary Tables**

**Supplementary Table S1:** Patients’ characteristics (n=68) at diagnosis and prior to allogeneic stem cell transplantation.

|  | **All patients,**  **n=68** |
| --- | --- |
| **Parameters at diagnosis** | |
| Sex, n (%)  female | 32 (47) |
| Disease origin, n (%)  *de novo* | 49 (72) |
| Hemoglobin, g/dL  median (range) | 8.9 (4.5-13.3) |
| Platelet count, x 10^9^/L  median (range) | 77 (2-305) |
| WBC, x 10^9^/L  median (range) | 9.3 (0.8-324) |
| Blood blasts, %  median (range) | 23 (0-95) |
| BM blasts, %  median (range) | 51 (20-95) |
| Normal karyotype, n (%)  present | 40 (61) |
| ELN2017 group, n (%)  favorable  intermediate  adverse | 24 (36)  16 (24)  27 (40) |
| *NPM1*, n (%)  mutated | 29 (43) |
| *CEBPA*, n (%)  mutated | 8 (12) |
| *FLT3*-ITD, n (%)  present | 10 (15) |
| *RUNX1*, n (%)  mutated | 11 (19) |
| *TP53*, n (%)  mutated | 2 (3) |
| DTA mutations, n (%)  all  *DNMT3A* mutated  *TET2* mutated  *ASXL1* mutated | 68 (100)  41 (62)  18 (33)  15 (22) |
| **Parameters prior to allogeneic HSCT** | |
| Age at HSCT, years  median (range) | 64.1 (34.7-75.3) |
| Remission status prior to HSCT  CR  CRi | 50 (74)  18 (26) |
| Donor type, n (%)  related, HLA matched  unrelated, HLA matched  HLA mismatched  haploidentical related | 9 (14)  38 (56)  19 (29)  2 (3) |
| Conditioning regimens, n (%)  MAC  RIC  NMA | 4 (6)  7 (10)  57 (84) |
| MRD at HSCT*,* n (%)  all  *NPM1*, positive  *BAALC*, positive  *MN1*, positive | 29 (57)  9 (38)  12 (27)  14 (32) |
| DTA mutations at HSCT*,* n (%)  all  *DNMT3A* mutated  *TET2* mutated  *ASXL1* mutated | 47 (85)  25 (86)  13 (93)  4 (67) |

*Abbreviations: ASXL1, additional sex combs-like 1 gene; BM, bone marrow; BAALC, brain and acute leukemia cytogenetic gene; CEBPA, CCAAT/enhancer-binding protein alpha gene; CR, complete remission; CRi, complete remission with incomplete peripheral recovery; DNMT3A, DNA methyltransferase 3 alpha gene; ELN, European LeukemiaNet; FLT3-ITD, internal tandem duplication in the FLT3 gene; Hb, hemoglobin; HLA, human leukocyte antigen; HSCT, hematopoietic stem cell transplantation MAC, myeloablative conditioning;; MN1, meningioma 1 gene; MRD, measurable residual disease; NMA, non-myeloablative conditioning; NPM1, nucleophosmin 1 gene; PB, peripheral blood; RIC, reduced intensity conditioning; RUNX1, Runt-related transcription factor 1 gene; TET2, Ten-Eleven Translocation-2 gene; TP53, tumor protein 53 gene; WBC, white blood count.*

**Supplementary Table S2.** Primer, probes, and PCR conditions for the analyzed canonical mutations in *DNMT3A* and *ASXL1*.

| **Mutation** | **Primer/Probes** | **PCR condition** | **Sensitivity** |
| --- | --- | --- | --- |
| *DNMT3A* R882 | cDNA  forward CCCATGTCCCTTACACAC  reverse GGTTTCCCAGTCCACTATAC  gDNA  forward GAAGAGGTGGCGGATGACT  reverse GGTTTCCCAGTCCACTATAC  wild type probe  R882C HEX - C[+C]AA[+G]CGG[+C][+T]C – BHQ1  R882H HEX - C[+C]AA[+G][+C][+G]GC[+T]C – BHQ1  mutation probe  R882C FAM – C[+C]A[+A]G[+C][+A][+G]C[+T]C – BHQ1  R882H FAM – CC[+A]A[+G][+T][+G]GCTCA – BHQ1 | 95°C 10 min  94°C 30 sec  60°C 2 min 40x  98°C 10 min  12°C hold | 0.0001 |
| *ASXL1* G646Wfs*12 | cDNA and gDNA  forward GAGGTCACCACTGCCATAGA  reverse CTGCCACCTCCCTCATCG  wild type probe  HEX - G[+A][+G]GGGGGG[+G][+T]GGC-BHQ1  mutation probe  FAM - A[+G]GGGGGGG[+G][+T]GGC-BHQ1 | 95°C 10 min  94°C 30 sec  52°C 2 min 60x  98°C 10 min  12°C hold | 0.0001 |

**Supplementary Table S3.** Overview of analyzed DTA mutations in CR/CRi prior to allogeneic HSCT per patient.

| **UPN** | ***DNMT3A*** | ***TET2*** | ***ASXL1*** |
| --- | --- | --- | --- |
| UPN 29 | R882H | - | - |
| UPN 83 | R882H | - | - |
| UPN 89 | - | - | G646Wfs*12 |
| UPN 106 | R882H | - | - |
| UPN 116 | - | K944* | - |
| UPN 143 | R882H | - | - |
| UPN 145 | - | R1452* | - |
| UPN 163 | R771fs | - | - |
| UPN 166 | R882C | - | - |
| UPN 186 | - | - | G646Wfs*12 |
| UPN 192 | R882C | - | - |
| UPN 206 | - | L1329R | - |
| UPN 207 | Q527* | - | - |
| UPN 218 | R882H | - | - |
| UPN 259 | R882H | - | - |
| UPN 291 | R882C | - | - |
| UPN 302 | W581R | - | - |
| UPN 324 | - | - | R693* |
| UPN 331 | - | R1452* | - |
| UPN 354 | E817* | - | - |
| UPN 360 | - | - | E657* |
| UPN 375 | G707D | - | - |
| UPN 383 | - | R1261H | - |
| UPN 388 | R882H | - | - |
| UPN 392 | W305* | - | - |
| UPN 395 | R882C | - | - |
| UPN 411 | S839T | - | - |
| UPN 412 | R882C | - | - |
| UPN 417 | R882H | - | - |
| UPN 424 | - | - | G646Wfs*12 |
| UPN 427 | G543D | - | - |
| UPN 440 | - | Q884* | G646Wfs*12 |
| UPN 447 | - | - | G646Wfs*12 |
| UPN 452 | P904L | Q1127Ifs*2 | - |
| UPN 461 | R882C | - | - |
| UPN 465 |  | Q1547* | - |
| UPN 466 | D531G | - | - |
| UPN 471 | - | T970I | - |
| UPN 479 | - | - | G646Wfs*12 |
| UPN 481 | - | - | G646Wfs*12 |
| UPN 497 | R882H | - | - |
| UPN 511 | R882H | R1404* | - |
| UPN 515 | - | Y1245* | - |
| UPN 518 | - | - | Q760* |
| UPN 519 | - | Q962* | - |
| UPN 525 | - | C1263R | - |
| UPN 599 | R882H | - | - |
| UPN 730 | R882C | - | - |
| UPN 804 | R882H | - | - |
| UPN 822 | - | L1872R | - |

*Abbreviations: UPN, unique patient number. Four patients had multiple TET2 mutations of which the one with the highest VAF at diagnosis was analyzed at HSCT. Additional TET2 mutations were present in UPN 145 (E628*), UPN 383 (E1215*), UPN 511 (I1873T), and UPN 511 (E1405D, P1460L, V1949A).*

**Supplementary Table S4.** Clinical, genetic and molecular information according to the persistence of a canonical *vs* non-canonical *DNMT3A* or *ASXL1* mutations at HSCT (n=39).

|  | **canonical *DNMT3A* or *ASXL1* mutation**  **n=26** | **non-canonical *DNMT3A* or *ASXL1* mutation**  **n=13** | ***P*** |
| --- | --- | --- | --- |
| **Parameters at diagnosis** | | | |
| Sex, n (%)  male  female | 12 (46)  14 (54) | 6 (46)  7 (54) | 1 |
| Disease origin, n (%)  *de novo*  secondary | 19 (73)  7 (27) | 10 (77)  3 (23) | 1 |
| Hemoglobin, g/dL  median (range) | 9.0 (4.5-12.8) | 8.1 (5.2-12.2) | .67 |
| Platelet count, x 10^9^/L  median (range) | 101 (15-305) | 32 (3-188) | .02 |
| WBC, x 10^9^/L  median (range) | 18.1 (2.1-324) | 37.6 (0.9-160) | .91 |
| Blood blasts, %  median (range) | 20 (0-95) | 28 (4-94) | .44 |
| BM blasts, %  median (range) | 50 (20-95) | 80 (28-95) | .51 |
| Normal karyotype, n (%)  present  absent | 17 (68)  8 (32) | 8 (62)  5 (38) | .73 |
| ELN2017 group, n (%)  favorable  intermediate  adverse | 11 (44)  5 (20)  9 (36) | 3 (23)  3 (23)  7 (54) | .46 |
| *NPM1*, n (%)  mutated  wild type | 16 (62)  10 (38) | 3 (23)  10 (77) | .04 |
| *CEBPA*, n (%)  mutated  wild type | 2 (8)  23 (92) | 0 (0)  13 (100) | .54 |
| *FLT3*-ITD, n (%)  present  absent | 7 (27)  19 (73) | 1 (8)  12 (92) | .23 |
| *RUNX1*, n (%)  mutated  wild type | 4 (21)  15 (79) | 4 (31)  9 (69) | .68 |
| *TP53*, n (%)  mutated  wild type | 0 (0)  19 (100) | 1 (8)  12 (92) | .41 |
| DTA mutations, n (%)  all  *DNMT3A* mutated  *TET2* mutated  *ASXL1* mutated | 26 (100)  19 (73)  2 (11)  7 (28) | 13 (100)  10 (77)  2 (15)  3 (23) | 1  1  1  1 |
| **Parameters at HSCT** | | | |
| Age at HSCT, years  median (range) | 65.5 (37.0-73.7) | 64.2 (52.0-75.3) | .53 |
| Remission status prior to HSCT  CR  CRi | 19 (73)  7 (27) | 12 (92)  1 (8) | .23 |
| Donor type, n (%)  related  unrelated, HLA matched  HLA mismatched | 3 (12)  13 (50)  10 (43) | 2 (15)  7 (54)  4 (31) | .90 |
| Conditioning regimens, n (%)  MAC  RIC  NMA | 2 (8)  0 (0)  24 (92) | 0 (0)  0 (0)  13 (100) | .54 |
| Pre-HSCT MRD*,* n (%)  positive  negative | 13 (54)  11 (46) | 10 (77)  3 (23) | .29 |
| Pre-HSCT clonal hematopoiesis*,* n (%)  all  *DNMT3A* mutated  *TET2* mutated  *ASXL1* mutated | 22 (85)  17 (89)  2 (100)  4 (57) | 10 (77)  8 (80)  1 (100)  1 (33) | .67  .59  1  1 |

*Abbreviations: ASXL1, additional sex combs-like 1 gene; BM, bone marrow; BAALC, brain and acute leukemia cytogenetic gene; CEBPA, CCAAT/enhancer-binding protein alpha gene; CR, complete remission; CRi, complete remission with incomplete peripheral recovery; DNMT3A, DNA methyltransferase 3 alpha gene; ELN, European Leukemia Net; FLT3-ITD, internal tandem duplication of the FLT3 gene; Hb, hemoglobin; HLA, human leukocyte antigen; HSCT, hematopoietic stem cell transplantation; MN1, meningioma 1 gene; MAC, myeloablative conditioninig; NMA, non-myeloablative conditioning; NPM1, nucleophosmin 1 gene; PB, peripheral blood; RIC, reduced intensity conditioning; RUNX1, Runt-related transcription factor 1 gene; TET2, Ten-Eleven Translocation-2 gene; TP53, tumor protein 53 gene; WBC, white blood count.*

**Supplementary Table S5.** Comparison of Bayesian Information Criterion (BIC) between MRD analysis at HSCT using a 3-marker model (*NPM1* mutation, *BAALC/ABL1* and *MN1/ABL1* expression) and a 5-marker model (*NPM1* mutation, *BAALC/ABL1* and *MN1/ABL1* expression as well as non-canonical *DNMT3A* and *ASXL1* mutations).

|  | **Cumulative Incidence of Relapse**  **BIC** | **Overall Survival**  **BIC** |
| --- | --- | --- |
| 3-marker model | 160.64 | 178.23 |
| 5-marker model | 160.59 | 174.25 |

**Supplementary Table S6.** Multivariate analyses.

|  | Cumulative Incidence of Relapse | | Overall Survival | |
| --- | --- | --- | --- | --- |
|  | **HR* (95% CI)** | ***P*** | **OR** (95% CI)** | ***P*** |
| ELN2017 genetic risk  (adverse *vs* intermediate *vs* favorable) | 1.85 (1.17-2.94) | .009 | - | - |
| MRD status at HSCT  (MRD positive *vs* negative) | 3.26 (1.38-7.67) | .007 | 0.25 (0.11-0.61) | .002 |
| *Abbreviations: CI, confidence interval; HSCT, hematopoietic stem cell transplantation; MRD, measurable residual disease.*  *HR, hazard ratio, <1 (>1) indicate lower (higher) risk of relapse for the first category listed for the dichotomous variables.  **OR, odds ratio, <1 (>1) indicate lower (higher) chance of survival for the first category listed for the dichotomous variables.  Variables considered in the models were those significant at α=0.10 in univariable analyses.  For cumulative incidence of relapse endpoint, variables considered were: ELN2017 genetic risk group, disease type (*de novo vs* secondary AML), number of remission (CR/CRi1 *vs* CR/CRi2), and MRD status at HSCT (based on *NPM1*, *BAALC/ABL1,* *MN1/ABL1* MRD and non-canonical *ASXL1* and *DNMT3A* mutation; positive *vs* negative). For OS endpoint, the only significant variable in univariate analysis was MRD status at HSCT. | | | | |

**Supplementary Figures**

**Supplementary Figure S1**

**Supplementary Figure S1. Outcome according to DTA mutations at diagnosis. (A)** Cumulative Incidence of Relapse and **(B)** Overall Survival in *DNMT3A vs TET2 vs ASXL1* mutated patients at diagnosis consolidated with an allogeneic HSCT (n=68).

**Supplementary Figure S2**

**Supplementary Figure S2.** Dilution series for four exemplary DTA mutations analyzed by ddPCR.

**Supplementary Figure S3**

**Supplementary Figure S3.** Correlation of VAF detection between NGS and ddPCR in DTA mutated AML patients.

**Supplementary Figure S4**

**Supplementary Figure S4. Prognostic significance of the 5-marker model (including *NPM1* mutations, *BAALC*/*ABL1*, and *MN1*/*ABL1* copy numbers and non-canonical *DNTM3A* and *ASXL1* mutation) based MRD status at HSCT in patients with DTA mutations at diagnosis (n=53). (A)** Cumulative incidence of relapse and **(B)** Overall survival according to positive *vs* negative MRD status for all markers combined, and **(C)** Cumulative incidence of relapse and **(D)** Overall survival according to the number of positive MRD tests.
